# Mre11 Assembles Linear DNA Fragments into DNA Damage Signaling Complexes

**DOI:** 10.1371/journal.pbio.0020110

**Published:** 2004-05-11

**Authors:** Vincenzo Costanzo, Tanya Paull, Max Gottesman, Jean Gautier

**Affiliations:** **1**Department of Genetics and Development, Columbia UniversityNew York, New YorkUnited States of America; **2**Department of Molecular Genetics and Microbiology, University of TexasAustin, TexasUnited States of America; **3**Institute of Cancer Research, Columbia UniversityNew York, New YorkUnited States of America

## Abstract

Mre11/Rad50/Nbs1 complex (MRN) is essential to suppress the generation of double-strand breaks (DSBs) during DNA replication. MRN also plays a role in the response to DSBs created by DNA damage. Hypomorphic mutations in *Mre11* (which causes an ataxia-telangiectasia-like disease [ATLD]) and mutations in the ataxia-telangiectasia-mutated (*ATM*) gene lead to defects in handling damaged DNA and to similar clinical and cellular phenotypes. Using *Xenopus* egg extracts, we have designed a simple assay to define the biochemistry of Mre11. MRN is required for efficient activation of the DNA damage response induced by DSBs. We isolated a high molecular weight DNA damage signaling complex that includes MRN, damaged DNA molecules, and activated ATM. Complex formation is partially dependent upon Zn^2+^ and requires an intact Mre11 C-terminal domain that is deleted in some ATLD patients. The ATLD truncation can still perform the role of Mre11 during replication. Our work demonstrates the role of Mre11 in assembling DNA damage signaling centers that are reminiscent of irradiation-induced foci. It also provides a molecular explanation for the similarities between ataxia-telangiectasia (A-T) and ATLD.

## Abstract 2

A defective cellular response to double-strand breaks in DNA is associated with several cancer-predisposition diseases. Specific defects affect the formation of a large protein complex that involves several key proteins

## Introduction

Cellular response to DNA damage requires the coordinated activation of cell cycle checkpoints with DNA repair (Zhou and Elledge 2000). Failure to block S-phase entry in response to damaged DNA or to repair the DNA leads to genomic instability, the hallmark of cancer cells. DNA double-strand breaks (DSBs) are particularly harmful to cells; if unrepaired, DSBs generate aneuploidy and chromosomal translocations. DSBs activate a network of signaling pathways that coordinate the sensing and repair of the damage with cell cycle arrest. The major signaling pathway triggered by DSBs involves ataxia-telangiectasia-mutated (ATM) protein kinase (Zhou and Elledge 2000). ATM is a serine-threonine kinase related to the PI3 kinase family. DSBs activate ATM by promoting its autophosphorylation (Bakkenist and Kastan 2003). Activated ATM phosphorylates protein substrates involved in DNA repair, cell cycle arrest, and apoptosis.

Phosphorylation of Nbs1 by ATM is critical for S-phase checkpoint (Gatei et al. 2000; Lim et al. 2000; Zhao et al. 2000). Nbs1 forms a trimeric complex with Mre11 and Rad50 (MRN) that is needed for DSB repair by homologous recombination (Haber 1998; D'Amours and Jackson 2002; Symington 2003). The three proteins are also essential for vertebrate embryonic development and cell growth (Luo et al. 1999; Yamaguchi-Iwai et al. 1999; Zhu et al. 2001). MRN prevents the accumulation of DSBs during DNA replication (Costanzo et al. 2001).

ATM function is defective in patients carrying the recessive genetic disorder ataxia-telangiectasia (A-T). A-T is characterized by cerebellar degeneration, immunodeficiency, radiation sensitivity, chromosomal instability, and cancer predisposition (Gatti et al. 2001).

Hypomorphic mutations in *Mre11* and *Nbs1* give rise, respectively, to an A-T-like disease (ATLD) and Nijmegen breakage syndrome (NBS) (Digweed et al. 1999; Stewart et al. 1999; Tauchi et al. 2002). The clinical presentations of A-T and ATLD are indistinguishable. NBS patients display the symptoms of A-T and ATLD and, in addition, microcephaly and mental deficiency (Tauchi et al. 2002). All three diseases have similar cellular phenotypes. The mutant cells do not respond appropriately to DSBs and display chromosome abnormalities, hypersensitivity to ionizing radiations, radio-resistant DNA synthesis, and an S-phase checkpoint defect (Shiloh and Kastan 2001). These similarities strongly suggest that ATM and MRN function in a common signaling pathway. However, the molecular connection between these proteins is yet to be determined.

Mre11 binds DNA and is both a 3′-to-5′ exonuclease and an endonuclease that cleaves hairpin DNA structures (Paull and Gellert 1999). Rad50 belongs to the structural maintenance of chromosomes (SMC) family of proteins. Rad50 contains C-terminal and N-terminal Walker A and B domains separated by a long coiled-coil domain (de Jager et al. 2001; Hopfner et al. 2002). Intramolecular assembly of the coiled-coil domain brings the Walker A and B motifs together to generate a functional nucleotide-binding module. A zinc-binding motif (CXXC), or “zinc hook,” located at the base of the Rad50 coiled coil, mediates Rad50 dimerization through coordination of a zinc ion by four cysteine residues (Hopfner et al. 2002).

Rad50 dimer binds to two Mre11 molecules to form a stable tetrameric complex with enhanced nuclease activities (Trujillo and Sung 2001). hRad50/hMre11 complexes tether linear duplex DNA molecules as demonstrated by scanning force microscopy (de Jager et al. 2001). Based on these observations, a model has been proposed in which the Mre11/Rad50 complex bridges broken DNA ends or sister chromatids (van den Bosch 2003).

In yeast and mammalian cells, DSBs provoke the formation of defined nuclear structures called irradiation-induced foci (IRIF). IRIF are believed to originate by chromatin modification, such as H2AX phosphorylation, at the site of the DSB, followed by the recruitment of signaling and repair factors. MRN localizes to DSBs, independently of H2AX phosphorylation, and is critical for the formation of IRIF and the consequent response to DNA damage (Petrini and Stracker 2003). Thus, cells with mutations in *Mre11* or *Nbs1* form IRIF inefficiently. In ATLD cells, which carry a defective *Mre11*, ATM activation is inhibited. Furthermore, ATM fails to localize to sites of DSBs in cells lacking functional MRN (Uziel et al. 2003). Taken together, these results suggest that MRN plays an early and essential role in assembly of functional signaling complexes at the sites of DNA damage. Furthermore, they place MRN upstream of ATM in the DNA damage signaling pathway.

Cell-free extracts derived from *Xenopus* eggs recapitulate signaling pathways triggered by DNA damage and have been instrumental in unraveling the functions of *ATM* and *Mre11* (Costanzo et al. 2000, 2001). Using this system, we show below that fragmented DNA assembles with proteins into macromolecular structures enriched in activated ATM and MRN. Their assembly requires MRN but not ATM. A truncated form of *Mre11* associated with ATLD does not support DNA–protein complex assembly or DSB-induced activation of ATM. This work provides a direct molecular connection between ATM and MRN that can explain the similarities between A-T and ATLD.

## Results

### A Rapid Assay for the Response to DNA DSBs

Addition of fragmented DNA to *Xenopus* egg extracts triggers the ATM-signaling pathway (Costanzo et al. 2000). We previously demonstrated this reaction by measuring an ATM-dependent block to DNA replication in extracts treated with DNA fragments (Costanzo et al. 2000). We now describe a rapid assay to monitor the activation of DSB-responsive protein kinases and to assess the contribution of ATM and related protein kinases. Histone H2AX, a well-characterized substrate for DSB-activated protein kinases, is phosphorylated in vivo at serine 139 by ATM and the ataxia-telangiectasia-related protein (ATR) (Rogakou et al. 1998; Burma et al. 2001; Costanzo et al. 2001; Ward and Chen 2001). We used the C-terminal peptide of mouse H2AX (PAVGKKA**S_134_**QA**S_139_**QEY) as a reporter substrate to monitor the response to DSBs. This peptide contains two putative SQ phosphorylation sites for ATM or ATR: serines 134 and 139. To test the specificity of the kinase(s) activated by DSBs, we synthesized four peptides: wild-type and alanine substitutions at serine 134 (S134A), serine 139 (S139A), and serines 134 and 139 (S134A/S139A).

**Figure 1:**
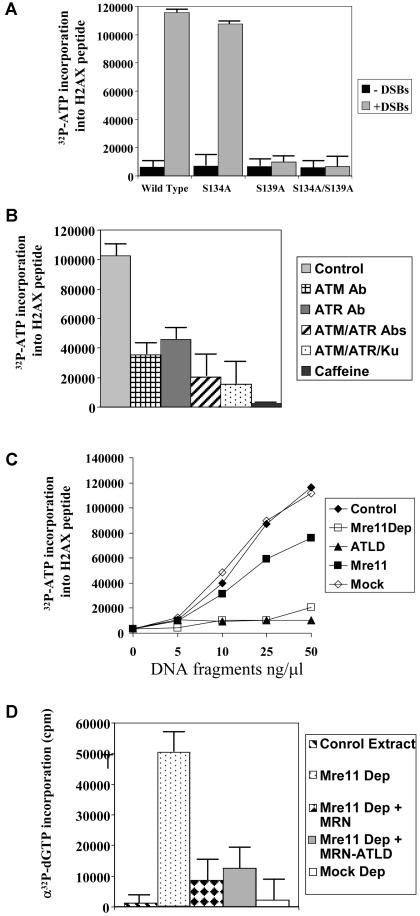
Functional MRN Is Required for the Response to DSBs, and Mre11–ATLD Separates Essential and Nonessential Mre11 Functions (A) The activity of protein kinases responsive to DSBs in Xenopus laevis egg extracts was monitored by incorporation of ^32^P from γ-^32^P-ATP into H2AX-derived peptides in the presence (plus DSB) or absence (minus DSB) of fragmented DNA. Labels: Wild-Type, H2AX substrate peptide containing serine 134 and serine 139; S134A, H2AX substrate peptide with a substitution of serine 134 to alanine; S139A, H2AX substrate peptide with a substitution of serine 139 to alanine; S134A/S139A, H2AX substrate peptide with a substitution of both serines to alanine. (B) Extract incubated with linear DNA at 50 ng/μl (equivalent to 4.5 × 10^10^ breaks/μl) was assayed with H2AX peptide in the presence of buffer (Control), ATM-neutralizing antibodies (ATM Ab), ATR-neutralizing antibodies (ATR Ab), ATM- and ATR-neutralizing antibodies (ATM/ATR Abs), ATM- and ATR-neutralizing antibodies in Ku70-depleted extracts (ATM/ATR Abs; Ku depletion), 5 mM caffeine (Caffeine). (C) DSB-responsive kinase activity was measured in the presence of 0, 5, 10, 25, and 50 ng/μl of linear DNA in control extract (filled diamonds), mock-depleted extract (open diamonds), Mre11-depleted extract (open squares), Mre11-depleted extract supplemented with 500 nM of recombinant MRN (filled squares), or Mre11-depleted extract supplemented with 500 nM MRN-ATLD1/2 (filled triangles). (D) DSB accumulation during DNA replication was monitored by TUNEL assay. Postreplicative nuclei were isolated from a control extract (stripes), Mre11-depleted extract (dots), Mre11-depleted extract supplemented with MRN (diamonds), Mre11-depleted extract supplemented with MRN-ATLD1/2 (gray) or mock-depleted extract (white).

Incubation of interphase extracts for 30 min with fragmented DNA dramatically enhanced phosphorylation of H2AX peptide (Figure 1A). Phosphorylated H2AX peptide could be detected as early as 5 min after addition of fragmented DNA (data not shown). S134A peptide was phosphorylated to a level equivalent to wild-type peptide, whereas S139A and S134/139A peptides were not modified. Thus, phosphorylation of S139 in cell-free extracts in response to DSBs mimics the in vivo situation (Rogakou et al. 1998; Burma et al. 2001; Costanzo et al. 2001; Ward and Chen 2001).

We next monitored phosphorylation of H2AX peptide in extracts in which specific DNA damage response signaling pathways were inhibited. X-ATM- and X-ATR-neutralizing antibodies were used to abrogate ATM- and ATR-dependent signaling, respectively. We previously demonstrated that these antibodies completely inhibit ATM- and ATR-dependent checkpoints in extracts (Costanzo et al. 2000, 2003). H2AX peptide phosphorylation was significantly reduced in extracts treated with either X-ATM or X-ATR antibodies. Inhibition of both ATR and ATM further decreased H2AX peptide phosphorylation to 20% of control levels (Figure 1B, column 4). Inhibition of DNA-PK by depletion of Ku70 did not further reduce H2AX peptide phosphorylation in the ATM/ATR-inhibited extract. Finally, caffeine completely abrogated H2AX peptide phosphorylation (Figure 1B, column 6). We conclude that most H2AX phosphorylation induced by DSBs in crude extracts is ATM- and ATR-dependent.

### Functional MRN Is Required for ATM Activation

Experiments using cells carrying hypomorphic mutations in *Nbs1* or *Mre11* (Carney et al. 1998; Varon et al. 1998; Stewart et al. 1999; Petrini and Stracker 2003) suggested that MRN also plays a role in sensing signals triggered by DSBs. However, because *Mre11* and *Nbs1* are essential genes (Yamaguchi-Iwai et al. 1999; Zhu et al. 2001; Tauchi et al. 2002), the effect of total Mre11 inactivation on the DNA damage response could not be established. We asked whether MRN was required in our system for DSB-dependent activation of H2AX peptide phosphorylation. We have previously established that Mre11 can be quantitatively depleted from extracts (Costanzo et al. 2001). Figure 1C shows that depletion of extracts for Mre11 abrogated the response to DSB-containing DNA (Figure 1C, open squares). Recombinant human MRN restored the DNA damage response in the Mre11-depleted extract (Figure 1C, filled squares).

ATLD, a syndrome characterized by failure of the DNA damage response, is caused by hypomorphic mutations in Mre11 (Stewart et al. 1999). In contrast to wild-type protein, MRN containing a mutant Mre11 that lacks the C-terminal DNA-binding domain (MRN-ATLD1/2) (Stewart et al. 1999; Lee et al. 2003) did not restore activity to the Mre11-depleted extract (Figure 1C, filled triangles).

At higher fragmented DNA concentrations (greater than or equal to 100 ng/μl), H2AX peptide phosphorylation became partly independent of ATM and Mre11. This phosphorylation was sensitive to vanillin, a specific inhibitor of DNA-PK (Durant and Karran 2003), and to Ku depletion (data not shown).

In contrast to its inactivity in the DSB checkpoint reaction, MRN-ATLD1/2 can fulfill the essential function of MRN in preventing the accumulation of breaks during DNA replication. Figure 1D shows a TUNEL assay to detect DNA ends. As we previously reported (Costanzo et al. 2001), chromosomal DNA replicated in Mre11-depleted extracts accumulated DSBs. Addition of purified recombinant MRN to depleted extracts largely prevented DNA fragmentation. MRN-ATLD1/2 was as efficient as wild-type MRN in supporting normal DNA replication.

These results establish that MRN is required to activate the DSB signal pathway and that the C-terminal region of Mre11 plays a critical role in this activation.

### Linear DNA Fragments Trigger Mre11-Dependent Assembly of Large DNA–Protein Complexes

**Figure 2:**
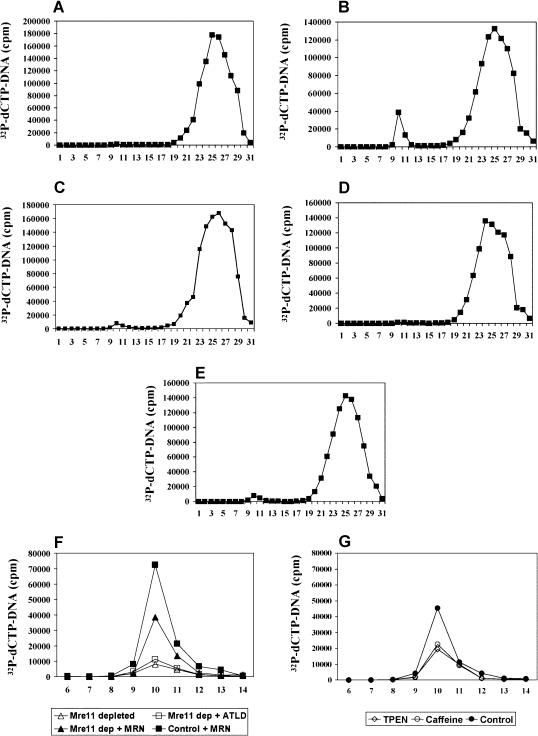
Requirements for the Assembly of DNA–Protein Complexes Elution profiles of α-^32^P-dATP-labeled 1 kb linear DNA from BioGel A15m chromatography columns. After loading, fractions 1–31 were collected and radioactivity was counted in a scintillation counter. (A–E) Complete elution profile. (A) Linear DNA alone. (B) Linear DNA incubated 2 h in extract at 22°C. (C) α-^32^P-dATP-labeled circular plasmid incubated for 2 h in extract at 22°C. (D) Linear DNA incubated with extract treated with 1 mg/ml proteinase K immediately prior to loading. (E) Linear DNA incubated in Mre11-depleted extract. (F and G) Excluded volume (fractions 6–14). (F) Linear DNA incubated in the following extracts: Mre11-depleted extract (open triangles), Mre11-depleted extract supplemented with 500 nM of MRN (filled triangles), Mre11-depleted extract supplemented with 500 nM of MRN-ATLD1/2 (open squares), or control extract supplemented with MRN (filled squares). (G) Linear DNA incubated in the following extracts: control extract (filled circles), extract treated with 5 mM caffeine (open circles), extract treated with TPEN at 100 μM (open diamonds).

Scanning force microscopy data (de Jager et al. 2001) show that Mre11–Rad50 binds preferentially to broken DNA ends, implying that direct interaction with linear DNA is essential for MRN function. To investigate interactions between Mre11 and damaged DNA, interphase extract was incubated with ^32^P-labeled, 1 kb linear double-strand DNA molecules and applied to a BioGel A15m column. This large-pore gel filtration resin includes most proteins and small DNA fragments, but excludes protein–DNA complexes larger than 1.5 × 10^7^ kDa (Yuzakhov et al. 1999). When radio-labeled DNA at the concentration of 50 ng/μl (equivalent to 4.5 × 10^10^ ends/μl) was applied to the column in the absence of extract (Figure 2A) or with extract but prior to incubation (data not shown), all radioactivity was recovered in the included volume. In contrast, when fragmented DNA was incubated with extract prior to chromatography, radio-labeled DNA resolved into two peaks (Figure 2B). Most DNA was still recovered in the included volume (fractions 20–30). However, a separate DNA peak corresponding to 3%–5% of the total DNA loaded appeared in the excluded volume (fractions 9–12). In contrast, labeled double-strand circular plasmid DNA did not assemble into DNA–protein complexes after incubation; all labeled DNA was recovered in the included volume (Figure 2C).

The peak in the excluded volume represents large DNA–protein complexes that assembled in the extract, since it was eliminated by treatment of the extract with proteinase K immediately prior to chromatography (Figure 2D). Note that the elution buffer contains detergent, ruling out possible membrane aggregation.

To determine whether Mre11 plays a role in assembling the DNA–protein complex, we incubated labeled DNA in an Mre11-depleted extract (Figure 2E). In the absence of Mre11, almost no radioactive label was recovered in the excluded volume. Addition of recombinant human MRN to the depleted extract restored the peak of high molecular weight DNA–protein complex (Figure 2F, filled triangles). We conclude, therefore, that the assembly of DNA–protein complexes requires Mre11. Furthermore, addition of MRN to nondepleted extract increased the amount of DNA in the excluded volume (Figure 2F, filled squares), suggesting that MRN is limiting in these extracts. MRN-ATLD1/2 did not restore DNA–protein complex formation in an Mre11-depleted extract (Figure 2F, open squares), indicating that an intact Mre11 C-terminal domain is required for complex assembly.

Rad50 protein forms intramolecular coiled-coil interactions as well as intermolecular interactions via a Zn^2+^-chelating hinge region coordinated by four cysteine residues, the “zinc-hook” (Hopfner et al. 2002). Addition of TPEN, a chelating agent specific for Zn^2+^ (Shumaker et al. 1998), partially inhibited formation of DNA–protein complexes (Figure 2G, open circles). Finally, caffeine significantly reduced but did not eliminate the amount of labeled DNA in the excluded peak (Figure 2G, open diamonds). This suggests that assembly of the DNA–protein complex is partially independent of ATM/ATR.

### MRN Complex Is Part of the DNA–Protein Complexes That Tethers Linear DNA Molecules

**Figure 3:**
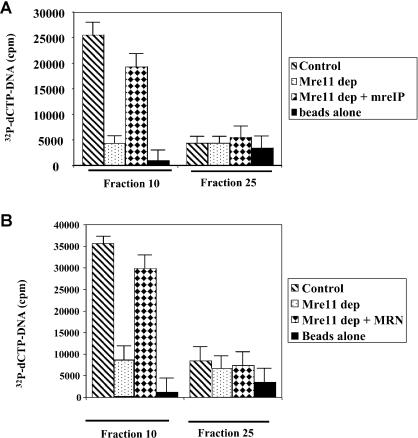
Mre11 Tethers DSB-Containing DNA (A) Control and treated extracts were incubated with α-^32^P-dATP-labeled DNA fragments and loaded onto BioGel A15 columns. Fractions 10 and 25 were collected and incubated with polyclonal antibodies against Mre11 or protein A beads alone. Beads were collected and washed, and radioactivity was counted in a scintillation counter. Shown are control extract (stripes), Mre11-depleted extract (dots), or Mre11-depleted extract supplemented with Mre11 that had been immunoprecipitated from the extract (diamonds), and extract incubated with beads alone (black). (B) Biotinylated DNA fragments were mixed with α-^32^P-dATP-labeled DNA fragments and incubated with various extracts. The extracts were then loaded onto BioGel A15 columns. Fractions 10 and 25 were collected and incubated with streptavidin-magnetic beads. Beads were collected and washed, and radioactivity was counted in a scintillation counter. Shown are control extract (stripes), Mre11-depleted extract (dots), Mre11-depleted extract supplemented with 500 nM MRN (diamonds), and streptavidin beads (black).

**Figure 4:**
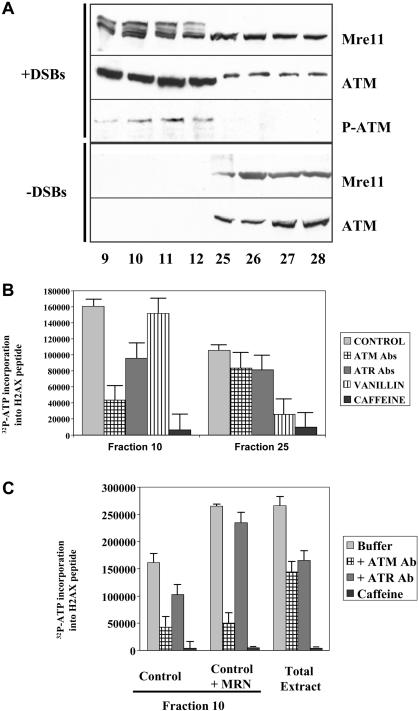
DNA–Protein Complexes Are Signaling Centers Containing Active Mre11 and ATM (A) Western blot analysis of eluted fractions. Fraction numbers are indicated at bottom. Fractions were collected following chromatography of extracts incubated with fragmented (plus DSBs) or without fragmented DNA (minus DSBs). Samples from fractions were processed for SDS-PAGE and blotted with polyclonal antibodies against Mre11, ATM, and phosphorylated ATM. (B) Activity of ATM and ATR kinases in fractions 10 and 25. Extracts were incubated with DNA fragments and applied to BioGel A15m columns. Fraction 10 and fraction 25 from control extract were assayed for H2AX activity in presence of buffer (light gray), ATM-neutralizing antibodies (checks), ATR-neutralizing antibodies (dark gray), 300 μM vanillin (stripes), or 5 mM caffeine (black). (C) Activity of ATM and ATR kinases in fraction 10 and total extract. Control extracts or extracts supplemented with 500 nM recombinant MRN were incubated with DSBs and loaded onto BioGel A15m columns. Total control extract and fraction 10 were assayed for H2AX activity in the presence of buffer (light gray), ATM-neutralizing antibodies (checks), ATR-neutralizing antibodies (dark gray), or 5 mM caffeine (black).

The previous experiments established that Mre11 is required for assembly of DNA–protein complexes. To demonstrate that Mre11 is an integral component of these complexes, we immunoprecipitated Mre11 from chromatographic fractions 10 and 25 and measured the ^32^P-DNA content of the precipitate. We found labeled DNA associated with MRN in fraction 10 (Figure 3A) but not in fraction 25, although this fraction contains both Mre11 (see Figure 4A) and ^32^P-DNA. As expected, immunoprecipitates of excluded fractions following chromatography of Mre11-depleted extracts did not contain labeled DNA. When Mre11 immunoprecipitated from control extracts was added to Mre11-depleted extract, we again found MRN–DNA complexes in fraction 10, but not in fraction 25 (Figure 3A).

We conclude that MRN is a component of the DNA–protein complex.

To demonstrate that linear DNA molecules were linked in the DNA–protein complex, we incubated extract with two populations of fragmented DNA. DNA molecules of identical sequence were either ^32^P-labeled or biotinylated. As expected, the radioactivity elution profile was identical to that observed with a single species of DNA molecules (data not shown). Fraction 10 from the excluded volume and fraction 25 from the included volume were precipitated with PEG, and biotinylated DNA molecules were affinity-purified using streptavidin-magnetic beads. The results of this assay show clearly that ^32^P-DNA was associated with biotinylated DNA in fraction 10, but not in fraction 25 (Figure 3B, columns 1 and 5). As expected, this association is Mre11-dependent. It was abolished in Mre11-depleted extracts and restored in Mre11-depleted extracts supplemented with recombinant MRN (Figure 3B, columns 2 and 3).

### Mre11-Containing DNA–Protein Complexes Are Signaling Centers

We next looked for a molecular connection between the DNA–protein complex and protein kinase activation. We monitored the distribution and modification of Mre11 and ATM in the chromatographic fractions described above. Control extracts or extracts incubated with fragmented DNA were chromatographed, and fractions 9–12 from the excluded volume and fractions 25–28 from the included volume were PEG-precipitated and processed for Western blotting. In untreated control extracts, Mre11 and ATM were recovered only in the included volume. In extracts treated with linear DNA, however, Mre11 and ATM were present in both included and excluded fractions (Figure 4A, top four panels). Relative to the total protein content of the fractions, both Mre11 and ATM were enriched 18-fold and 46-fold respectively in the excluded fraction, as determined by image analysis. This confirms that the high molecular weight protein–DNA complexes contain Mre11, and additionally establishes the presence of ATM in the complex. Strikingly, Mre11 in the DNA–protein complex was in the active, phosphorylated form (Costanzo et al. 2001). In contrast, Mre11 in the included fractions was unmodified.

Furthermore, using an antibody that recognizes specifically the active form of ATM (phosphorylated on the serine equivalent to serine 1,981 of human ATM; Bakkenist and Kastan 2003), we detected phosphorylated ATM only in the excluded fractions (Figure 4A, third panel).

To confirm that the excluded peak was enriched in active ATM kinase, we compared H2AX peptide kinase activity in fractions 10 and 25. We also determined the relative contribution of ATM, ATR and DNA-PK to this activity (Figure 4B). ATM and ATR protein kinase activities were inhibited with the specific neutralizing antibodies described above. DNA-PK activity was inhibited by vanillin, a specific inhibitor of DNA-PK (Durant and Karran 2003). Most H2AX kinase activity in fraction 10 was due to ATM, and to a lesser extent, to ATR (Figure 4B). Vanillin had little effect on kinase activity, indicating that the contribution of DNA-PK was small in this fraction. In contrast, the kinase activity in fraction 25 was sensitive to vanillin, but not to ATM- or ATR-neutralizing antibodies (Figure 4B).

To provide further evidence that formation of MRN–DNA complexes directly promotes ATM activation, we supplemented extracts with recombinant MRN and compared H2AX peptide phosphorylation in total extract and in fraction 10. The proportion of H2AX kinase that was inhibited by ATM antibodies was significantly higher in fraction 10 than in total extract (compare columns 1 and 2 with columns 9 and 10 in Figure 4C). Incubation of extract with recombinant MRN complex prior to chromatography increased H2AX kinase activity in fraction 10 by 80% (compare columns 1 and 5 in Figure 4C). The increased kinase activity was entirely abrogated by anti-ATM antibody (Figure 4C, columns 2 and 6).

## Discussion

### MRN Complex Is Required for ATM Activation

The three components of the MRN complex, Mre11, Rad50, and Nbs1, are essential. Mouse embryos or chicken cells carrying inactivating mutations in any of these proteins are not viable (Luo et al. 1999; Yamaguchi-Iwai et al. 1999; Zhu et al. 2001). This has made studies of MRN and its interacting partners difficult to approach. Although a connection between ATM activation and MRN has long been known (Petrini 2000), the precise mechanism that links these two factors had not, to our knowledge, been elucidated. However, using cell-free *Xenopus* egg extracts, it has been possible to inactivate biochemically essential gene products. We previously determined that depletion of Mre11 and its associated protein partners lead to DSB formation during DNA replication (Costanzo et al. 2001). We used a similar strategy to relate MRN inactivation and ATM function.

We provide several lines of evidence that indicate an MRN requirement for ATM activation. The G1–S checkpoint provoked by DSBs entails the sequential activation of protein kinases, including ATM (Zhou and Elledge 2000). We show that depletion of Mre11 from our extracts abolishes DSB-dependent phosphorylation of H2AX peptide, a readout for this cascade. ATM is the major contributor to H2AX phosphorylation in these extracts. Our data strongly suggest that MRN specifically activates ATM. Fragmented DNA incubated in extracts forms high molecular weight DNA–protein complexes that include MRN and ATM. Of H2AX kinase activity in the complex in fraction 10, 75% is inhibited by antibodies to ATM. Furthermore, addition of recombinant MRN to extracts increases the yield of complex and associated H2AX kinase activity. The enhanced activity is entirely ATM-dependent.

ATR also contributes significantly to H2AX phosphorylation in extracts treated with DSB-containing DNA. However, ATM is activated earlier than ATR (data not shown). ATR activation might be triggered by processing of DSBs into single-strand DNA (ssDNA) (Zou and Elledge 2003)**.** We previously showed that ssDNA specifically stimulates ATR (Costanzo et al. 2003). Since Mre11 depletion completely prevents H2AX phosphorylation, we propose that Mre11 regulates both ATM-dependent early signaling from DSBs and, possibly by its DNA exonucleolytic activity, delayed signaling by ATR. Whereas caffeine completely inhibits H2AX kinase, treatment with ATM/ATR antibodies combined inhibits only 80% of H2AX kinase. This could be accounted by an additional kinase such as ATX (Abraham 2001). Alternatively, the neutralizing antibodies against ATM and ATR might not inhibit 100% of the activity of respective kinase towards H2AX.

### MRN Tethers Linear DNA Molecules and Assembles DNA Damage Signaling Complexes

We propose that MRN interacts with linear DNA to form DNA–protein complexes that induce the phosphorylation cascade responsible for the G1–S checkpoint. MRN assembles with linear DNA molecules in vitro (de Jager et al. 2001). We have isolated DNA–protein complexes from extracts incubated with fragmented DNA as an excluded fraction from a sizing column. The complexes require Mre11 for assembly, contain linear DNA, and are highly enriched in Mre11 and ATM. Immunoprecipitation studies with Mre11 antibodies show the presence of tripartite complexes (Mre11–ATM–fragmented DNA) in the excluded but not the void volume (data not shown).

We believe that the formation of these complexes is a critical step in the kinase cascade that leads to the G1–S checkpoint. Several lines of evidence support this idea: (1) Mre11-depleted extracts do not form complexes and fail to activate ATM in response to DSBs. (2) Mre11 is concentrated 18-fold in the DNA–protein complexes and is heavily phosphorylated. We previously established that phosphorylation of Mre11 correlates with increased nuclease activity (Costanzo et al. 2001). (3) ATM is enriched 46-fold in the complexes and is phosphorylated on serine 1,981 (Bakkenist and Kastan 2003). Therefore, activated ATM is only detected in the DNA–protein complexes.

ATM, and possibly ATR, participates in the assembly of the complexes. Pretreatment of extracts with caffeine, an inhibitor of ATM and ATR, significantly reduces the yield of complex.

Some H2AX kinase activity is not associated with the DNA–protein complex. This activity is principally accounted for by DNA-PK.

Both MRN components Mre11 and Nbs1 are phosphorylated in response to DSBs. Nbs1 phosphorylation is ATM-dependent (Gatei et al. 2000; Lim et al. 2000; Zhao et al. 2000). Once recruited and activated within the signaling complex, ATM might phosphorylate Nbs1 and Mre11, stabilizing the complex and enhancing signaling activity. How might DNA–MRN complexes initiate the cascade of events leading to ATM activation? One of the critical steps could be to bring ATM in close proximity with “chromatinized” DNA fragments. Indeed, it was shown previously that ATM had affinity for DSBs (Andegeko et al. 2001; Uziel et al 2003). ATM enrichment at sites of DSBs is consistent with the localized phosphorylation of H2AX observed in vivo on chromatin flanking DSBs (van den Bosch et al. 2003).

Our previous work showed that at high doses of DNA fragment (100 ng/μl, equivalent to 9 × 10^10^ breaks/μl), the ATM-dependent checkpoint does not require Mre11 function (Costanzo et al. 2001). We also determined that H2AX phosphorylation at 100 ng/μl of linear DNA is partially Mre11-independent (data not shown). This could be due to ATM activation by mass action at this dose of linear DNA as well as to activation of DNA-PK (data not shown).

### Molecular Bases for the Similarities between A-T and ATLD

A powerful argument for placing MRN and ATM in a common signaling pathway derives from the similarities between the clinical and the cellular phenotypes of A-T, NBS, and ATLD (Digweed et al. 1999; Stewart et al. 1999; Tauchi et al. 2002). Uziel et al. (2003) recently showed that the ATM response to DSBs is impaired in ATLD cells, which carry defective Mre11. After our work was completed, additional studies reached similar conclusions using Mre11- or Nbs1-deficient cells (Carson et al. 2003; Mochan et al. 2003; Theunissen et al. 2003). Our data provide a biochemical framework to explain their observations.

The ATLD1/2 mutation, which generates a truncated Mre11 that lacks part of its DNA-binding domain, is compatible with viability. Thus, the mutation cannot abrogate the essential role of Mre11, although the mutant Mre11 is defective in the damaged DNA response. We were able to dissociate the two Mre11 reactions using simple biochemical readouts. MRN-ATLD1/2 cannot activate ATM or form DNA–protein complexes in response to DSBs. It can, however, prevent accumulation of DSBs during chromosomal DNA replication. We speculate that MRN-ATLD1/2 has reduced affinity for damaged DNA, resulting in labile interactions with fragmented DNA and an inability to activate ATM.

What differentiates the essential function of Mre11 during DNA replication from its ability to activate ATM? We suggest that MRN association with chromatin during DNA replication and, possibly, during meiotic recombination differs from its association with fragmented DNA. Consistent with this hypothesis, chromatin association of Mre11 was shown, by detergent extraction, to differ between replicative and γ-irradiated chromatin (Mirzoeva and Petrini 2003). We previously demonstrated the association of Mre11 with chromatin during normal DNA replication. One can envisage MRN complexes forming on intact chromatin in a manner similar to other SMC proteins such as cohesins, and involving, perhaps, interactions with cohesins (Kim et al. 2002). These complexes could perform the essential functions of MRN during replication and recombination and would not require an intact Mre11 C-terminal domain. This is consistent with the viability and recombination proficiency of ATLD mutant cells.

In contrast, tethering of damaged DNA containing DSBs would require the Mre11 C-terminal DNA-binding domain. Failure to interact with broken DNA would account for the various phenotypes of A-T and ATLD.

Alternatively, C-terminal truncation of Mre11 might weaken protein–protein interactions within the MRN complex or between MRN and other proteins. This idea is suggested by the Mre11 crystal structure, which shows that the C-terminal domain in close proximity to a hydrophobic region required for protein–protein interaction (Hopfner et al. 2001). The truncated Mre11 might be unable to form the protein–protein interactions required to stabilize MRN–DNA complexes.

### MRN–DNA Complexes and IRIF

The signaling complexes described above are reminiscent of IRIF observed in mammalian cells (Maser et al. 1997). Indeed, Mre11 is one of the first proteins to localize to IRIF following DNA damage (Petrini and Stracker 2003). Furthermore, cells from ATLD patients fail to establish foci (Stewart et al. 1999), consistent with the inability of MRN-ATLD1/2 to support the formation of DNA–protein complexes in extracts. Recall that the ability to form foci and to activate a DNA damage response in mammalian cells are closely correlated (Stewart et al. 1999, 2003; Goldberg et al. 2003).

There are several similarities between the formation of IRIF in vivo and assembly of the signaling structures in extracts. Both require (1) intact Mre11 protein and, presumably, binding of Mre11 to DNA, and (2) that IRIF form independently of (Mirzoeva and Petrini 2001), but are stabilized by, ATM, possibly by phosphorylation of Mre11 and/or Nbs1 (Gatei et al. 2000; Lim et al. 2000; Wu et al. 2000; Zhao et al. 2000; Costanzo et al. 2001; Lukas et al. 2003).

**Figure 5:**
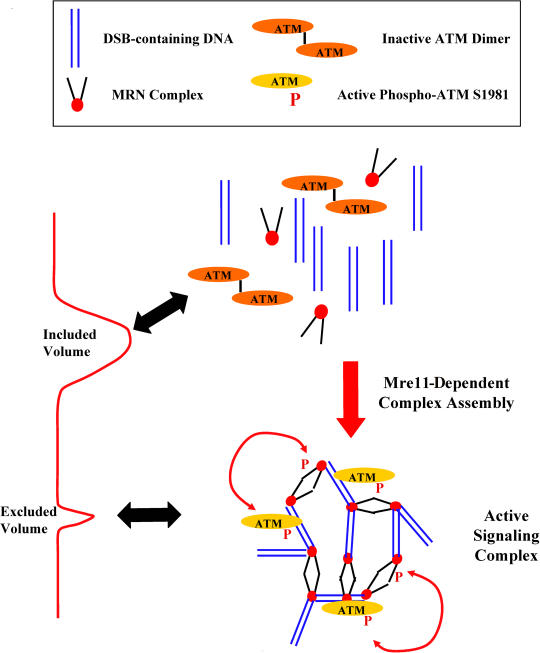
Schematic Representation of the Mre11-Dependent Assembly of DNA Damage Signaling Complexes MRN promotes the assembly of DNA–protein structures containing linear DNA fragments enriched with active ATM molecules. These active signaling complexes resemble IRIF in that they are the morphological and functional unit of the DNA damage response.

As shown in Figure 5, our data suggest that MRN concentrates and localizes DNA fragments and signaling proteins such as ATM in IRIF-like structures. MRN may be rate-limiting for assembly of these structures, even though Mre11 can be recovered apart from DNA–protein complexes. It was recently reported that the ends of broken chromosomes localize with phosphorylated H2AX to discrete spots in the nucleus (Aten et al. 2004). The formation of these structures requires functional MRN. We suggest that these are the in vivo counterparts of the MRN-dependent structures that we observe in vitro. We have shown that DNA–protein complexes are essential for the DNA damage checkpoint. The challenge now is to dissect the assembly pathway and to identify the rate-limiting steps in the organization of these signaling centers.

## Materials and Methods

### 

#### *Xenopus* egg extracts

CSF-arrested extracts were freshly prepared according to Costanzo et al. (2001). For kinase assays, extracts were supplemented with 100 mg/μl cycloheximide and released into interphase with 0.4 mM CaCl_2_.

#### DNA template

To prepare DNA fragments containing DSBs, we used pBR322 plasmid digested with restriction endonucleases to yield different types of ends (3′-overhang, 5′-overhang, and blunt). These DNA fragments behaved equivalently in our assay (data not shown). For the experiments shown in Figure 1, we used DNA digested with HaeIII.

The 1 kb DNA fragment used for size fractionation experiments was obtained by PCR on M13 ssDNA template using 22 nt primers complementary to positions 5,570 and 6,584 (de Jager et al. 2001). The ^32^P-labeled fragment was obtained by addition of α-^32^P-dATP (10 mCi/μl) to the PCR. The biotinylated 1 kb fragment was obtained by PCR on M13 ssDNA template using a 22 nt primer complementary to position 5,570 and a 22 nt primer complementary to position 6,584, biotinylated on three thymidine residues (Sigma-Genosys, The Woodlands, Texas, United States).

#### Kinase assays

Interphase egg extracts were incubated with DNA fragments, DNA fragments and ATM-neutralizing Ab, ATR-neutralizing antibodies or 5 mM caffeine for 30 min at 22°C. Extract (2 μl) was mixed with 20 μl of EB kinase buffer (20 mM HEPES [pH 7.5], 50 mM NaCl, 10 mM MgCl_2_, 1 mM DTT, 1 mM NaF, 1 mM Na_3_VO_4_, and 10 mM MnCl_2_) supplemented with 0.5 mg/ml histone H2AX peptide (Sigma-Genosys), 50 μM ATP, and 1 μl of γ-^32^P-ATP, 10 mCi/μl (greater than 3,000 Ci/mmol). Samples were incubated at 30°C for 20 min, and reactions were stopped by 20 μl of 50% acetic acid and spotted on p81 phosphocellulose filter paper (Upstate Biotechnology, Lake Placid, New York, United States). Filters were air-dried and washed three times in 10% acetic acid. Radioactivity was quantified in a scintillation counter.

For kinase assays of fractionated extracts, 50 ng/μl of 1 kb DNA fragments was incubated in interphase extracts at 22°C for 2 h. Extracts were loaded onto the sizing column, and 250 μl fractions were collected. Fractions were supplemented with 9% PEG-6000, incubated on ice for 15 min, and spun in a microfuge at maximum speed at 4°C for 10 min. Pellets were resuspended in 20 μl of EB buffer, and 2 μl was assayed with histone H2AX peptide substrate, with or without ATM-neutralizing antibodies, ATR-neutralizing antibodies, 300 μM vanillin, or 5 mM caffeine.

#### Egg extract fractionation

Interphase egg extracts (200 μl) were incubated with or without 50 ng/μl of ^32^P-labeled 1 kb DNA fragments for 2 h at 22°C. They were then mixed with one volume of buffer A, loaded onto a 15 × 300 mm column prepacked with BioGel A15m resin (Bio-Rad, Hercules, California, United States) previously equilibrated with buffer A at 4°C. Extracts were mock-depleted, Mre11-depleted, or Mre11-depleted supplemented with 500 nM MRN or with 500 nM MRN-ATLD1/2. Control extracts were treated with 500 nM MRN and 100 μM TPEN or 5 mM caffeine or 1 mg/ml proteinase K at 37°C. After the samples were loaded, 15 ml of buffer A (100 mM KCl, 40 mM HEPES [pH 8.0], 0.05% Tween-20, 10 mM MgCl_2_, 1 mM ATP, 1 mM DTT, 1 mM NaF, 1 mM Na_3_VO_4_ leupeptin, pepstatin, and aprotinin protease inhibitors) were gently applied to the column. We collected 31 fractions of approximately 300 μl, and radioactivity was measured in a scintillation counter. For the elution profile of the circular plasmid in control extracts, a 1.8 kb plasmid derived form pUC19 with the SspI–SapI region deleted (Ristic et al. 2001) was used. Nicked plasmid was isolated and labeled by nick translation in the presence of α-^32^ P-dCTP, ligated, and incubated in extracts. After the fractions were collected, radioactivity was counted in a scintillation counter.

#### Precipitation of DNA fragments bound to Mre11

We incubated 200 μl of control, Mre11-depleted, or Mre11-depleted extract supplemented with Mre11 precipitated from the extract with 50 ng/μl of ^32^P-labeled 1 kb DNA fragments. Samples were applied to the BioGel A15m sizing column and fractions were collected. Void volume peak fraction 10 and included volume peak fraction 25 were incubated with 50 μl of specific polyclonal antibodies against Mre11 prebound to protein A–Sepharose beads or beads alone overnight at 4°C . Beads were washed with buffer A, and radioactivity was counted in a scintillation counter.

#### Biotinylated DNA pulldown

We incubated 200 μl of control, Mre11-depleted, or Mre11-depleted extract supplemented with 500 nM MRN with 50 ng/μl ^32^P-labeled 1 kb DNA fragments and 50 ng/μl biotinylated 1 kb fragments for 2 h at 22°C. Samples were applied to the BioGel A15m sizing column, and fractions were collected. Void volume peak fraction 10 and included volume peak fraction 25 were incubated with kilobase-BINDER dynabeads (Dynal Biotech, Oslo, Norway) or mock protein A dynabeads (Dynal Biotech), and DNA fragments were isolated according to the kit protocol. Biotinylated DNA fragments bound to beads were washed with buffer A, and radioactivity was counted in a scintillation counter.

#### **Recombinant Mre11/Rad50/Nbs1Proteins**

Human MRN and MRN-ATLD1/2 were purified from baculovirus-infected cells according to published protocols (Paull and Gellert 1998). The recombinant trimeric complex was used at a concentration of 500 nM, unless otherwise specified.

#### X-**Mre11 complex depletion/Ku depletion**

For X-Mre11 complex depletion, 50 μl of interphase extract was incubated with 25 μl of protein A–Sepharose beads coupled with 50 μl of preimmune serum or with 50 μl of X-Mre11 antiserum for 60 min at 4°C. For Ku70/80 depletion, 50 μl of interphase extract was incubated with 25 μl of protein A–Sepharose beads coupled to 50 μl of Ku antiserum (Covance, Princeton, New Jersey, United States) for 60 min at 4°C.

#### **TUNEL assay**

TUNEL assay was performed according to Costanzo et al. (2001).

#### Western blot

We incubated 2 μl samples of interphase egg extracts for 30 min at 22°C with 50 ng/μl DNA fragments, DNA fragments and 5 mM caffeine, or with 50 ng/μl circular plasmid, and 2 μl samples were recovered from the BioGel A15m column and precipitated with 9% PEG (see Figure 3A) were diluted in loading buffer, boiled for 3 min, electrophoresed on 6% or 10% SDS-PAGE, transferred to nitrocellulose, and probed with polyclonal antibodies specific for *Xenopus* ATM, *Xenopus* ATR, *Xenopus* Mre11, phosphoserine 1,981 of human ATM (Rockland Immunochemicals, Gilbertsville, Pennsylvania, United States), and phosphorylated ATM/ATR SQ substrates (New England Biolabs, Beverly, Massachusetts, United States).

## Abbreviations

A-T: ataxia-telangiectasia
ATLD: ataxia-telangiectasia-like disease
ATM: ataxia-telangiectasia mutated
ATR: ataxia-telangiectasia-related
DSB: double-strand break
IRIF: irradiation-induced foci
MRN: Mre11/Rad50/Nbs1 complex
MRN-ATLD1/2: Mre11/Rad50/Nbs1 complex containing truncated Mre11
NBS: Nijmegen breakage syndrome
SMC: structural maintenance of chromosomes
ssDNA: single-strand DNA
