# Correction: Mre11 Assembles Linear DNA Fragments into DNA Damage Signaling Complexes

**DOI:** 10.1371/journal.pbio.0020229

**Published:** 2004-06-15

**In *PLoS Biology*, volume 2, issue 5:**

Mre11 Assembles Linear DNA Fragments into DNA Damage Signaling Complexes

**Vincenzo Costanzo, Tanya Paull, Max Gottesman, Jean Gautier**

DOI: 10.1371/journal.pbio.0020110

Because of a labeling error, the size of the DNA fragment used throughout the experiments was reported incorrectly (reported as 1 kb). The actual size of the DNA fragment was 131 bp. The fragment corresponds to the M13 DNA sequence from nucleotide 5656 to nucleotide 5787. This difference in fragment size does not affect any of the conclusions of the paper.

This correction note may be found online at DOI: 10.1371/journal.pbio.0020229.

